# COVID-19 Cases and Transmission in 17 K–12 Schools — Wood County, Wisconsin, August 31–November 29, 2020

**DOI:** 10.15585/mmwr.mm7004e3

**Published:** 2021-01-29

**Authors:** Amy Falk, Alison Benda, Peter Falk, Sarah Steffen, Zachary Wallace, Tracy Beth Høeg

**Affiliations:** ^1^Department of Pediatrics, Aspirus Doctors Clinic, Wisconsin Rapids, Wisconsin; ^2^Medical College of Wisconsin-Central Wisconsin, Wausau, Wisconsin; ^3^ReVision Eye Care, Wisconsin Rapids, Wisconsin; ^4^University of California, Davis; ^5^Northern California Orthopaedic Associates, Sacramento, California.

The coronavirus disease 2019 (COVID-19) pandemic has disrupted in-person learning in the United States, with approximately one half of all students receiving online-only instruction since March 2020.* Discontinuation of in-person schooling can result in many hardships (*1*) and disproportionately affects families of lower socioeconomic status (*2*). Current evidence suggests that transmission of SARS-CoV-2, the virus that causes COVID-19, in kindergarten through grade 12 (K–12) schools might not significantly contribute to COVID-19 spread nationwide (*3*). During August 31–November 29, 2020, COVID-19 cases, spread, and compliance with mask use were investigated among 4,876 students and 654 staff members who participated in in-person learning in 17 K–12 schools in rural Wisconsin. School-attributable COVID-19 case rates were compared with rates in the surrounding community. School administration and public health officials provided information on COVID-19 cases within schools. During the study period, widespread community transmission was observed, with 7%–40% of COVID-19 tests having positive results. Masking was required for all students and staff members at all schools, and rate of reported student mask-wearing was high (>92%). COVID-19 case rates among students and staff members were lower (191 cases among 5,530 persons, or 3,453 cases per 100,000) than were those in the county overall (5,466 per 100,000). Among the 191 cases identified in students and staff members, one in 20 cases among students was linked to in-school transmission; no infections among staff members were found to have been acquired at school. These findings suggest that, with proper mitigation strategies, K–12 schools might be capable of opening for in-person learning with minimal in-school transmission of SARS-CoV-2.

Among 18 selected schools in Wood County, Wisconsin, 17 agreed to participate in this study of COVID-19 in schools and compliance with mask use. One school opted not to participate based on teacher preference. Surveillance was initiated by a small group of physician and medical student researchers. Participating schools were from three public school districts, one private school district, and one independent private school. Eight schools were elementary (grades K–6) with 1,529 students attending in-person, and nine were secondary (grades 7–12) with 3,347 students attending in-person. An estimated 12.4% of Wood County’s children were attending virtually.

A number of infection mitigation measures were employed at the schools. The Legacy Foundation of Central Wisconsin provided funding for the districts to purchase 2–3-layer cloth face coverings for all students, and all students received three to five masks as a result of this grant. All schools were under district and statewide mask mandates during the study period. Students were asked to wear masks when within 6 feet of another person outdoors and at all times indoors. A classroom cohort included students from one grade level who avoided mixing with other students and ranged in size from 11 to 20 students. All classes and lunch periods were held indoors. Schools generally attempted to seat students near the same person within their cohort, if possible. Staff members were instructed to wear masks, maintain a distance of 6 feet from all persons, if possible, and limit time in shared indoor spaces. If a student was excluded from in-person school because of COVID-19 symptoms, that student’s siblings also were excluded from school. No systematic COVID-19 screening was conducted in the schools or the community.

A free online survey using Google Forms (https://www.google.com/intl/en-GB/forms/about) was distributed to all eligible classroom teachers (305) by the school administration or the research team. Information regarding the total number of students expected to attend school in-person, number of students actually attending in-person, and number of students donning or wearing masks when expected to do so was obtained from these surveys. Teachers were instructed to complete the survey once per week during a single class and were instructed to complete the survey based on what they were observing at that time on survey day. Information on masking compliance among staff members was not collected.

Information was obtained from the Wood County public health COVID-19 dashboard^†^ on weekly cases and percentage of positive COVID-19 test results in the community. A COVID-19 case was defined as a positive SARS-CoV-2 reverse transcription–polymerase chain reaction (RT-PCR) test result. COVID-19 cases in schools were reported by public health or school administration officials using deidentified data. Infection source and whether the infection was likely acquired in school or outside of school were determined by case investigations conducted by school administration and the public health department. When a school was alerted to a positive case in a student or staff member, school officials identified persons who had had close contact with the patient through interviews with the patient, parents, and school staff members. Close contact was defined as being within 6 feet for longer than 15 cumulative minutes during a 24-hour period.^§^ Patients’ close contacts were required to quarantine in their homes, and if they experienced symptoms during the quarantine period, they were further investigated to determine whether in-school spread might have occurred.

Descriptive statistics were used to calculate school and district average masking compliance as well as percentage of students absent based on the weekly surveys. The protocol was reviewed by the Aspirus Wausau Hospital Institutional Review Board and determined to be exempt from human subjects review because it met the requirements under 45 CFR 46. 104 (d) (2) and underwent a limited review as required under 46.111 (a) (7).

A total of 4,876 students and 654 staff members contributed data to the study. Wood County in central Wisconsin has a population of approximately 73,000, with just under 100 persons per square mile. According to a 2019 U.S. Census Bureau estimate,^¶^ 92.0% of the population in Wood County identified as non-Hispanic White, median income was $54,913, and 10.7% of persons met poverty thresholds.** During the 13-week study period (August 31–November 29), a total of 3,393 COVID cases were reported in Wood County (cumulative incidence = 5,466 per 100,000 persons), including 191 cases within the participating schools (cumulative incidence = 3,454 per 100,000). Cases occurred in 133 students and 58 staff members. Among these 191 cases, seven (3.7%) were attributed to in-school SARS-CoV-2 transmission (Figure 1), and all occurred among students. Five cases of transmission occurred within elementary school cohorts, and two occurred within secondary school cohorts. Three of these seven cases occurred in one class in one elementary school, and the other four occurred at separate schools. No in-school transmission between separate classroom cohorts was reported. Weekly COVID-19 incidence ranged from 34 to 1,189 per 100,000 persons in the community, and from 72 to 699 cases per 100,000 among students and staff members in the schools. COVID-19 incidence in schools conducting in-person instruction was 37% lower than that in the surrounding community. During the study period, 7%–40% of RT-PCR tests from Wood County had positive results (Figure 2).

**FIGURE 1:**
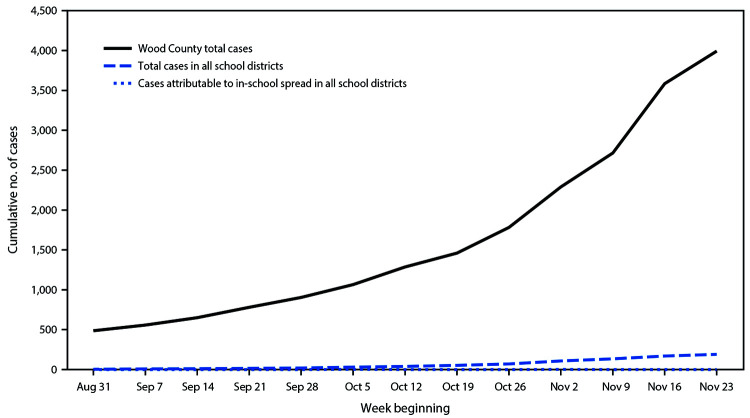
Cumulative number of community and school-associated* COVID-19 cases and in-school transmission,^†^ by week — Wood County, Wisconsin, August 31–November 29, 2020 **Abbreviation:** COVID-19 = coronavirus disease 2019. * Cases occurring in students or school staff members. ^†^ Cases attributed to virus transmission occurring during students’ attendance at schools.

**FIGURE 2:**
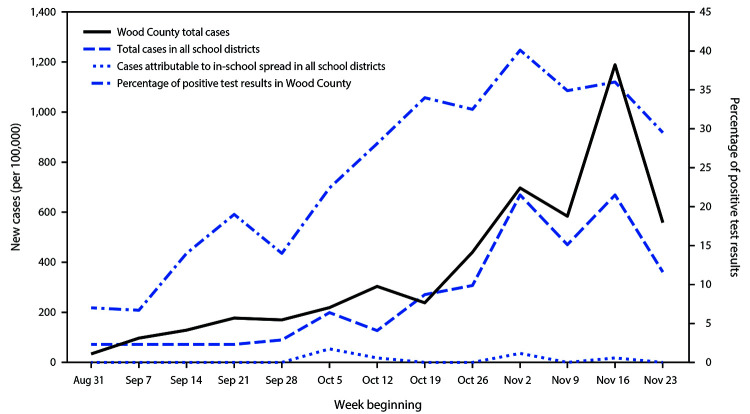
Community and school-associated COVID-19 incidence (cases per 100,000) and percentage of positive test results, by week — Wood County, Wisconsin, August 31– November 29, 2020 **Abbreviation:** COVID-19 = coronavirus disease 2019.

A total of 2,846 teacher survey responses were collected weekly (response rate = 54%), including 37,575 weekly student masking observations. Observed student masking compliance ranged from 92.1% to 97.4% (Figure 3) and did not vary by student age. During the study period, masking noncompliance increased slightly from 2.6% to 7.9%.

**FIGURE 3:**
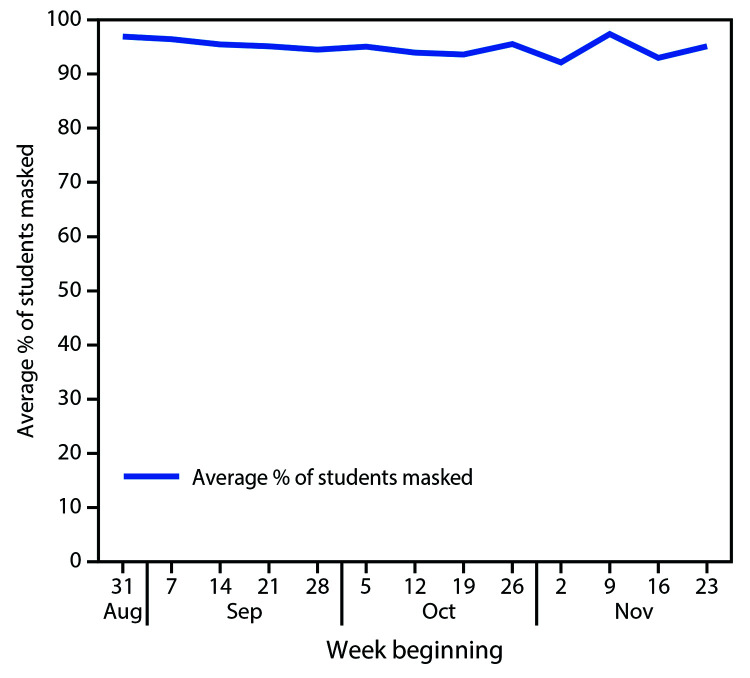
Average percentage of students (N = 4,876) in compliance with recommended mask use across all districts — Wood County, Wisconsin, August 31–November 29, 2020

## Discussion

This study, involving students and staff members in 17 K–12 schools in five rural Wisconsin districts under district and statewide mask mandates, found high teacher-reported student masking compliance. Among 5,530 students and staff members, 191 COVID-19 cases were reported. Only seven (3.7%) of these cases were associated with in-school transmission, all in students. Despite widespread community transmission, COVID-19 incidence in schools conducting in-person instruction was 37% lower than that in the surrounding community.

Children might be more likely to be asymptomatic carriers of COVID-19 than are adults (*4*). In the present study, the absence of identified child-to-staff member transmission during the 13-week study period suggests in-school spread was uncommon. This apparent lack of transmission is consistent with recent research (*5*), which found an asymptomatic attack rate of only 0.7% within households and a lower rate of transmission from children than from adults. However, this study was unable to rule out asymptomatic transmission within the school setting because surveillance testing was not conducted.

Student masking compliance was reported to exceed 92% throughout the course of the study. Older children were reported to be equally compliant with masking as younger children. High levels of compliance, small cohort sizes (maximum of 20 students), and limited contact between cohorts likely helped mitigate in-school SARS-CoV-2 transmission and could be responsible for the low levels of transmission detected in schools. Investigation of 191 school-related COVID-19 cases in students and staff members suggested that most transmission occurred outside of required school activities. This finding is consistent with recently reported data suggesting limited transmission within schools (*6*).

Some school districts throughout the country have set thresholds for reopening based on the percentage of positive test results in the community (e.g., Virginia: 10%, California: 8%) (*7*,*8*). The percentage of positive COVID-19 test results ranged from 7% to 40% in the community, and confirmed COVID-19 cases within schools were few. These findings suggest that attending school where recommended mitigation strategies are implemented might not place children in a higher risk environment than exists in the community. Having children in a monitored school setting might increase adherence to mask compliance, and cohorting can help minimize exposures for children and adults. In-person schooling for children has numerous health and societal benefits, especially for children and parents of lower socioeconomic status (*9*).

The findings in this report are subject to at least seven limitations. First, mask use was assessed using a survey that was not validated, dependent on voluntary teacher response and subject to recall and social desirability biases (*10*). The actual mask-wearing rate might have been different because only approximately one half of teachers participated in the study. Teachers with lower masking compliance in their cohort might have been less likely to complete the survey, which limits the reliability of this measure. Second, lack of data about masking compliance among staff members might also lead to a reported masking compliance that differed from actual masking compliance among all persons in the study. Third, it was not possible to determine the specific roles that mask-wearing and other disease mitigation strategies played in the low rate of disease spread, and information on school ventilation systems was not obtained. Fourth, because schools did not perform infection screening of staff members and students, the prevalence of asymptomatic spread could not be determined. However, recent serological survey data from a school setting found asymptomatic spread to be minimal.^††^ Fifth, sources of infection among identified cases were detected through contact tracing, which is less accurate than is genomic sequencing. Sixth, rural schools might differ in important ways from those in more densely populated areas. For example, the capacity to achieve physical distancing in schools might differ if classroom size and outdoor space in rural schools is different from that in suburban or urban schools. However, all the classes and lunch periods in this study were held indoors, as would be consistent with most urban settings. Finally, the ethnic makeup of this rural population was predominantly non-Hispanic White, and the results of this study might not be generalizable to other rural or nonrural school populations.

In a setting of widespread community SARS-CoV-2 transmission, few instances of in-school transmission were identified among students and staff members, with limited spread among children within their cohorts and no documented transmission to or from staff members. Only seven of 191 cases (3.7%) were linked to in-school transmission, and all seven were among children. Mask-wearing among students was reported by teachers as high, which likely contributed to low levels of observed disease transmission in these 17 K–12 schools. Although asymptomatic transmission is possible, this study demonstrated that, with precautions in place, in-school transmission of SARS-CoV-2 appeared to be uncommon in this rural Wisconsin community, despite up to a 40% positive SARS-CoV-2 test rate in the surrounding county.

**Summary**

**What is already known about this topic?**

COVID-19 outbreaks related to kindergarten through grade 12 (K–12) classroom settings have been rarely reported; however, in-school transmission risk has not been well described.

**What is added by this report?**

Among 17 rural Wisconsin schools, reported student mask-wearing was high, and the COVID-19 incidence among students and staff members was lower than in the county overall (3,453 versus 5,466 per 100,000). Among 191 cases identified in students and staff members, only seven (3.7%) cases, all among students, were linked to in-school spread.

**What are the implications for public health practice?**

With masking requirements and student cohorting, transmission risk within schools appeared low, suggesting that schools might be able to safely open with appropriate mitigation efforts in place.

## Footnotes

* Accessed January 13, 2021. https://cai.burbio.com/school-opening-tracker/

† Accessed December 10, 2020. https://woodwi.maps.arcgis.com/apps/opsdashboard/index.html#/da7f0d6815494e4b85e614e042671b14

§ CDC has defined “close contact” at the following URL: https://www.cdc.gov/coronavirus/2019-ncov/global-covid-19/operational-considerations-contact-tracing.html#:~:text=Close%20contact%20is%20defined%20by,the%20patient%20is%20isolated

¶
https://www.census.gov/quickfacts/woodcountywisconsin

**
https://aspe.hhs.gov/poverty-guidelines
